# Supplementary material for: Model based analysis of the heterogeneity in the tumour size dynamics differentiates vemurafenib, dabrafenib and trametinib in metastatic melanoma
Source: Cancer Chemother Pharmacol. 2017 Dec 8;81(2):325–32. doi: 10.1007/s00280-017-3486-3 (PMC5778167; doi:10.1007/s00280-017-3486-3)
Supplement: Supplementary file 1 — Supplementary material 1 (DOCX 28 KB) [file 280_2017_3486_MOESM1_ESM.docx]

**Supplementary Information**

**Model Development Tables**

Tables S1, S2 and S3 go through the step-wise improvements in the log-likelihood as the piece-wise linear model was developed. A cross in the Fixed Effects column corresponds to whether that parameter is included in a model e.g. Model 1 across all studies is simply a constant model. A cross in the IL Random Effects column corresponds to treating lesions as independent from each other, whereas a cross in the PT and IL in PT Random Effects column corresponds to accounting for which patient the lesions belong to when specifying the distribution for a given parameter.

For each structural model (constant, linear, piece-wise linear) we assessed the difference in likelihood between accounting for which lesion belongs to which patient versus treating lesions as they are independent from each other. In all cases knowing which lesion belongs to which patient gave a favourable change in the log-likelihood. During the development of the Vemurafenib model we noticed on several occasions that the variance on a certain parameter shrunk to zero. This indicates that a distribution on that parameter was not required and indeed when we removed the distribution on that parameter the log-likelihood did not change. For example compare models 3 and 4 in Table S1. They show that if using a linear model to fit to the data adding a distribution on *d* makes no difference to the log-likelihood.

Table S1 Table shows the development of the final model for Vemurafenib. The final model is highlighted in green. The IL random effects column corresponds to the first part of the analysis, not accounting for which lesion belongs to which patient, whereas the IL in PT column does.

| Vemurafenib | Fixed Effects | | | IL Random Effects | | | PT and IL in PT Random Effects | | | SP | Log-Likelihood | Notes |
| --- | --- | --- | --- | --- | --- | --- | --- | --- | --- | --- | --- | --- |
|  | BSL | d | g | BSL | d | g | BSL | d | g |  |  |  |
| Model 1 | x |  |  | x |  |  |  |  |  |  | -9841 |  |
| Model 2 | x |  |  |  |  |  | x |  |  |  | -9750 |  |
| Model 3 | x | x |  | x | x |  |  |  |  |  | -9800 | variance on d shrinks to 0 |
| Model 4 | x | x |  | x |  |  |  |  |  |  | -9800 |  |
| Model 5 | x | x |  |  |  |  | x |  |  |  | -9731 |  |
| Model 6 | x | x |  |  |  |  | x | x |  |  | -9623 |  |
| Model 7 | x | x | x | x | x | x |  |  |  | 63 | -9793 | variance on d and g shrinks to 0 |
| Model 8 | x | x | x | x | x | x |  |  |  | 116 | -9677 | variance on g shrinks to 0 |
| Model 9 | x | x | x | x | x | x |  |  |  | 179 | -9731 | variance on g shrinks to 0 |
| Model 10 | x | x | x | x |  |  |  |  |  | 63 | -9793 |  |
| Model 11 | x | x | x | x | X |  |  |  |  | 116 | -9677 |  |
| Model 12 | x | x | x | x | x |  |  |  |  | 179 | -9731 |  |
| Model 13 | x | x | x |  |  |  | x | x | x | 63 | -9386 | variance on g shrinks to 0 |
| Model 14 | x | x | x |  |  |  | x | x | x | 116 | -9548 | variance on g shrinks to 0 |
| Model 15 | x | x | x |  |  |  | x | x | x | 179 | -9609 | variance on g shrinks to 0 |
| Model 16 | x | x | x |  |  |  | x | x |  | 63 | -9386 | final model |
| Model 17 | x | x | x |  |  |  | x | x |  | 116 | -9548 |  |
| Model 18 | x | x | x |  |  |  | x | x |  | 179 | -9609 |  |

Table S2 Table shows the development of the final model for Dabrafenib. The final model is highlighted in green. The IL random effects column corresponds to the first part of the analysis, not accounting for which lesion belongs to which patient, whereas the IL in PT column does.

| Dabrafenib | Fixed Effects | | | IL Random Effects | | | PT and IL in PT Random Effects | | | SP | Log-Likelihood | Notes |
| --- | --- | --- | --- | --- | --- | --- | --- | --- | --- | --- | --- | --- |
|  | BSL | d | g | BSL | d | g | BSL | d | g |  |  |  |
| Model 1 | x |  |  | x |  |  |  |  |  |  | -6681 |  |
| Model 2 | x |  |  |  |  |  | x |  |  |  | -6672 |  |
| Model 3 | x | x |  | x | x |  |  |  |  |  | -6568 |  |
| Model 4 | x | x |  |  |  |  | x | x |  |  | -6545 |  |
| Model 5 | x | x | x | x | x | x |  |  |  | 63 | -6435 |  |
| Model 6 | x | x | x | x | x | x |  |  |  | 116 | -6499 |  |
| Model 7 | x | x | x | x | x | x |  |  |  | 179 | -6566 |  |
| Model 8 | x | x | x |  |  |  | x | x | x | 63 | -6404 | final model |
| Model 9 | x | x | x |  |  |  | x | x | x | 116 | -6477 |  |
| Model 10 | x | x | x |  |  |  | x | x | x | 179 | -6531 |  |

Table S3 Table shows the development of the final model for Trametinib. The final model is highlighted in green. The IL random effects column corresponds to the first part of the analysis, not accounting for which lesion belongs to which patient, whereas the IL in PT column does.

| Trametinib | Fixed Effects | | | IL Random Effects | | | PT and IL in PT Random Effects | | | SP | Log-likelihood | Notes |
| --- | --- | --- | --- | --- | --- | --- | --- | --- | --- | --- | --- | --- |
|  | BSL | d | g | BSL | d | g | BSL | d | g |  |  |  |
| Model 1 | x |  |  | x |  |  |  |  |  |  | -6179 |  |
| Model 2 | x |  |  |  |  |  | x |  |  |  | -6164 |  |
| Model 3 | x | x |  | x | x |  |  |  |  |  | -6073 |  |
| Model 4 | x | x |  |  |  |  | x | x |  |  | -6031 |  |
| Model 5 | x | x | x | x | x | x |  |  |  | 63 | -5972 |  |
| Model 6 | x | x | x | x | x | x |  |  |  | 116 | -6004 |  |
| Model 7 | x | x | x | x | x | x |  |  |  | 179 | -6057 |  |
| Model 8 | x | x | x |  |  |  | x | x | x | 63 | -5899 | final model |
| Model 9 | x | x | x |  |  |  | x | x | x | 116 | -5968 |  |
| Model 10 | x | x | x |  |  |  | x | x | x | 179 | -6018 |  |

**Final Model Parameter Values**

|  | log(BSL (mm) ) | | | d  (mm/day) | | | g  (mm/day) | | | SP (Days) |
| --- | --- | --- | --- | --- | --- | --- | --- | --- | --- | --- |
|  | mean | s.d. (BP) | s.d. (WP) | mean | s.d. (BP) | s.d. (WP) | mean | s.d. (BP) | s.d. (WP) |  |
| Vemurafenib  Value  (95% CI) | 3.38  (3.3, 3.46) | 0.41  (0.35, 0.48) | 0.34 (0.30, 0.37) | -0.26  (-0.33, -0.20) | 0.38  (0.34, 0.44) | 0.32  (0.29, 0.35) | 0.02 (0.003, 0.04) | NA | NA | 63 |
| Dabrafenib  Value  (95% CI) | 3.25  (3.19, 3.31) | 0.26 (0.19, 0.34) | 0.40 (0.36, 0.44) | -0.24  (-0.26, -0.21) | 0.11 (0.08, 0.14) | 0.11 (0.09, 0.14) | 0.06 (0.04, 0.08) | 0.09 (0.06, 0.14) | 0.07 (0.04, 0.10) | 63 |
| Trametinib  Value  (95% CI) | 3.22 (3.16, 3.29) | 0.26 (0.20, 0.34) | 0.42 (0.39, 0.47) | -0.13 (-0.15, -0.11) | 0.09 (0.07, 0.11) | 0.13 (0.11, 0.15) | 0.05 (0.03, 0.07) | 0.12 (0.09, 0.15) | 0.03 (0.01, 0.07) | 63 |

Table S4 Parameter estimates for the between patient (BP) and within patient (WP) distributions, mean and standard deviation (s.d.), for each parameter in the final model, for each drug with 95% confidence intervals.
